# Development of an inventory of goals using the International Classification of Functioning, Disability and Health in a population of non-ambulatory children and adolescents with cerebral palsy treated with botulinum toxin A

**DOI:** 10.1186/s12887-017-0974-x

**Published:** 2018-01-04

**Authors:** Linda Nguyen, Ronit Mesterman, Jan Willem Gorter

**Affiliations:** CanChild Centre for Childhood Disability Research, Department of Pediatrics, McMaster University, 1400 Main Street West, Room 408, Hamilton, ON L8S 1C7 Canada

**Keywords:** Botulinum toxin A, Child, Cerebral Palsy, Family-centred care, Needs assessment, International Classification of Functioning, Disability and Health

## Abstract

**Background:**

In the management of hypertonicity in children with cerebral palsy (CP), goals should be clearly identified in order to evaluate the effectiveness of botulinum toxin A (BoNT-A) treatment, specifically in non-ambulatory children and adolescents, Gross Motor Function Classification System (GMFCS), level IV or V. A retrospective chart review (Mesterman et al., 2013) identified the need for the development of a set of specific and meaningful goals linked to the International Classification of Functioning, Disability and Health (ICF) for future goal setting and evaluation in this population. Our objective is to create an inventory of goals based on the ICF framework that captures the needs and values of families with children with CP.

**Methods:**

This cross-sectional observational study recruited parents of twenty children and youths with CP in GMFCS levels IV or V (mean age 11.2 years, SD 4.3, 13 males) who were assessed for BoNT-A treatment at the Spasticity Management Clinic at McMaster Children’s Hospital (Hamilton, ON). A previous inventory of goals was developed by a group of experts at a national botulinum toxin conference held in January 2014 (Montreal, Canada). The inventory of goals was further refined by asking the parents to select goals from the inventory list that they would like their child to accomplish after receiving BoNT-A treatment, and asking healthcare professionals for clarity and phrasing of goals in the inventory list.

**Results:**

All parents identified body structure and function goals, with more than 75% of parents selecting reduction in muscle tone and increased range of movements in the upper and lower extremities. More than 50% of parents identified activity goals related to ease of caregiving. Two activity goals and three participation goals were missing from the inventory. Participation goals were identified by less than 5% of parents.

**Conclusion:**

The inventory may be a helpful tool to facilitate a discussion about goal setting between healthcare professionals and families in the context of BoNT-A treatment. A future study is needed to conduct qualitative interviews to better understand the information that families may require about setting goals during BoNT-A treatment and to evaluate the usefulness of the inventory.

**Electronic supplementary material:**

The online version of this article (10.1186/s12887-017-0974-x) contains supplementary material, which is available to authorized users.

## Background

Cerebral Palsy (CP) describes a group of chronic disorders affecting the patients’ development of movement and posture, and causing activity limitation [1]. Children with CP can be classified according to the Gross Motor Function Classification System (GMFCS), which consists of five levels of function, from level I (least affected) to level V (most affected) [2]. Botulinum Toxin A (BoNT-A) injections are widely used as a treatment to reduce muscle tone, which can help to improve joint range of motion [3]. There have been two international consensus papers stating that the use of BoNT-A treatment may be effective in reducing muscle tone and improving range of muscle movement [4, 5].

Historically, the treatment was directed at improving impairment such as increased tone, but ultimately improving functional ability. Treatment programs are now focusing on incorporating the International Classification of Functioning, Health, and Disability (ICF) model when managing children and youth with CP [6]. Assessment, treatment, and evaluation will not only focus on “body function and structure” but could include “activity” and “participation” as well as domains of caregiver assistance in daily living.

In order to make informed decisions about treatment, it is important for healthcare professionals to incorporate the perspectives of the patient and the family. Family-centred services offer an approach to provide information and support for parents [7]. It allows healthcare professionals to address family needs, and contribute to increased satisfaction in healthcare services for the family [8–10]. The goal setting process provides an opportunity for parents and healthcare professionals to have a discussion and identify goals during treatment [11, 12]. This will help determine the desired outcomes that parents and healthcare professionals would like to achieve. Parents and children are more likely to follow-up on treatment decisions if their perspectives are incorporated into the decision-making process [13]. Healthcare providers would also be able to better understand how they could provide quality and appropriate services that would meet the needs of the family.

Rehabilitation plans need to be comprehensive and encompass family needs. However, in clinical settings, goals are often not well documented [14, 15]. The framework of goals linked to the ICF has been shown to be useful [14, 15]. In the population of children with CP, GMFCS levels IV and V, it is not always clear what goals parents have for their child in the context of BoNT-A treatment. A former retrospective study reviewed the charts of 60 children diagnosed with CP, classified as GMFCS levels IV or V, who had received BoNT-A treatment [16]. The identified goals were jointly decided by the clinic staff and the patient and their family, and were grouped by the ICF domains into: body structure and function; activity and participation; and self-care. Goals were clearly identifiable in 80% of the charts, and were achieved in 75-80% of the cases. Due to the nature of the retrospective study, it was not always clear whether the goals were set by the clinician, parents, or both clinician and parents.

At a national botulinum toxin conference held in Montreal in January 2014, a special interest group (SIG) of physicians with experience in BoNT-A treatment identified goal setting in the context of BoNT-A treatment as a priority but a challenge for children with CP. At the meeting, the SIG developed a draft list of potential goals that might be relevant to families and children diagnosed with CP across all five GMFCS levels [17]. This list of goals is still a work-in-progress, and a comprehensive list needs to be developed that captures family values.

This study aimed to further develop and refine the draft of the inventory of goals that was developed based on the healthcare provider input at the national botulinum toxin conference [17]. In this study, we addressed the following question: What are the goals that have been identified by parents of children with CP, GMFCS levels IV or V, between 0 and 18 years who have received BoNT-A treatment? Our ultimate goal is to create an inventory of goals based on the ICF framework that captures the needs and values of families with children with CP.

## Methods

### Participants

In this observational study, a convenience sample of parents with children with CP was recruited at the Spasticity Management Clinic at McMaster Children’s Hospital. It is a multidisciplinary clinic comprised of two physicians, one nurse, two physiotherapists, one occupational therapist, and a child life specialist. The inclusion criteria to participate in this study were: 1) any child diagnosed with CP, GMFCS levels IV or V, who is considered for BoNT-A injections; 2) patient of the Spasticity Management Clinic at McMaster Children’s Hospital. This study received ethics approval from the Hamilton Integrated Research Ethics Board, Hamilton, Canada. Assent forms were offered, but the parents indicated that none of the children were capable of giving assent. All parents completed an informed consent form.

### Subject characteristics

There were 20 parents who were recruited as participants from January 2015 to April 2015. Three individuals who were approached did not participate due to inconvenience and lack of time. Participants were recruited until no new additional goals were provided, and data saturation was reached. Data saturation was continuously assessed by the student research assistant who recorded field notes of the frequency and types of goals that were raised by parents. Data saturation was identified to be reached based on a discussion with the research team, and sufficient information was acquired to demonstrate common goals that were frequently identified by parents in the clinic.

The characteristics of subjects (i.e. children and adolescents) are presented in Table 1. Sixty nine percent of subjects classified as levels IV or V according to the Communication Function Classification System (CFCS). In addition, 70% of subjects were classified as levels IV or V according to the Manual Ability Classification System (MACS). Cognitive impairments were assessed informally by the healthcare professional team. Children and adolescents were identified to have cognitive impairments based on their level of understanding during discussions in clinical appointments, such as their processing level of information, responsiveness to questions, and communication with healthcare professionals. All subjects were identified to have cognitive impairments, which may affect their ability to understand and set goals in a discussion with their parents and healthcare professional team.

**Table 1:** Subject characteristics

Characteristics	Subjects, *N* = 20
Age (in years	
mean ± SD	11.2 ± 4.3
Age range	5 -17
Gender, n (%)	
Male	13 (65)
Female	7 (35)
GMFCS, n (%)	
level IV	10 (50)
level V	10 (50)
MACS, n (%)	
level I	1 (5)
level II	2 (10)
level III	3 (15)
level IV	5 (25)
level V	9 (45)
^a^CFCS, *n* = 19, n (%)	
level I	3 (16)
level II	2 (10)
level III	1 (5)
level IV	6 (32)
level V	7 (37)
Cognitive impairments	20 (100%)

^a^Data unavailable for one participant

### Development of the inventory of goals

**Fig. 1:**
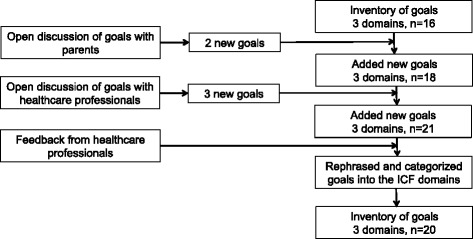
Development of the inventory list of goals in the context botulinum toxin treatment

We developed the inventory of goals in the context of BoNT-A using two steps: feedback from parents of children diagnosed with CP, and feedback from therapists in the Spasticity Management Clinic (See Fig. 1).

The initial inventory of goals was developed based on the ideas raised of clinicians with expertise at the national botulinum toxin conference held in January 2014 (Montreal, Canada). It consisted of 16 items in three domains based on the ICF framework: body structure/function, activity, and participation. The goals were specific to children and adolescents with CP and receiving BoNT-A treatment. Some of these goals were identified by parents in a retrospective study and achieved in 75-80% of cases [16].

Parents would select each item that was relevant and meaningful to the family, and that they would like their child to accomplish after receiving botulinum toxin treatment. Parents would indicate their selection of goals by checking off the box beside each item. After selecting goals in the inventory, parents were provided with the opportunity to add new goals that were not listed in the inventory.

The initial inventory of goals was provided to the healthcare professionals at the Spasticity Management Clinic for feedback. The team of healthcare professionals included one nurse, two physiotherapists, one occupational therapist, and one child life specialist with over five years of experience. The healthcare professionals were also asked for the additions of new goals that were missing from the inventory, phrasing and clarity of the goals, and the categorization of goals in the ICF framework.

### Study design

Children and their families were invited to participate in this study while attending their regular clinic appointment at the Spasticity Management Clinic at McMaster Children’s Hospital. Both parents were invited to participate in the interview. Assessment in clinic was conducted as usual, but there was a more thorough discussion on setting goals related to BoNT-A treatment. The interviews took place after the assessment by the therapist, but before a decision was made with the physician. After a decision was made and BoNT-A treatment was recommended, it was administrated either in clinic or under sedation at a different time.

For the purpose of this study, the interview was conducted with parents during the clinical appointment. Initially parents were asked an open-ended question of: “What kinds of goals do you have for your child with cerebral palsy after receiving botulinum toxin treatment?” Following the discussion, parents were provided with a draft of the inventory of goals developed by SIG (see Additional file 1, PDF document: Inventory of Goals in the Context of Botulinum Toxin A Treatment). Parents were asked to identify and select the goals listed in the inventory that they find are most important or meaningful to achieve following the BoNT-A treatment. After completing the inventory of goals, parents were asked to provide additional goals that they had considered and were missing from the inventory. The family, child, and multidisciplinary team also had a discussion about the process of setting goals when the child was being assessed for BoNT-A treatment. The goals discussed and selected by the family and the spasticity team members were recorded by the student research assistant who was observing the discussion.

Treatments goals were categorized according to the domains of body function/structure, activity, and participation in the International Classification of Functioning, Disability, and Health (ICF) [7]. The categorization of the goals into the ICF domains was based on a discussion between the research assistant and healthcare professionals in the Spasticity Clinic at McMaster Children’s Hospital.

### Data analysis

All data collected from parents were recorded into an Excel spreadsheet. The number of goals that was raised by parents was identified through frequency counts. The mean number of goals among parents of children with CP, classified as GMFCS levels IV or V, was calculated. The differences in goals between children with CP, classified as GMFCS levels IV and V, MACS levels, and CFCS levels were further explored using descriptive statistics. Comments and suggestions from healthcare professionals about BoNT-A treatment goals were included into the inventory by adding goals that were initially missing from the inventory, modifying the wording of the goals, and categorizing the goals into the ICF domains.

## Results

### Involvement of parents in goal setting

For each child, there was only one parent identifying the goals who was considered to be the primary caregiver by the family. When parents were first provided with the inventory of goals, they were unclear about the process of goal setting. Some parents felt overwhelmed and burdened by the aspect of goal setting because they questioned whether healthcare professionals, such as the clinicians and therapists, had already identified appropriate goals. Some parents would also ask about the types of goals that they should have for their child with CP. There were two parents with children with CP, GMFCS level IV, who hoped that the treatment would “fix” their child and selected all of the goals in the inventory that they wanted their child to achieve.

More than 75% of parents wanted BoNT-A treatment to help manage body structure and function, specifically reduce muscle tone. All began by identifying body structure and function goals. They required prompts in order to initiate a discussion about how BoNT-A treatment could help their child in achieving activity and participation goals. When parents selected activity goals from the inventory list, they were focused on how they could better care for their child. Parents further stated that they had challenges in transferring their child into different positions and would like the BoNT-A treatment to help facilitate ease in transfers.

Most parents had difficulties in identifying participation goals. Some parents commented how their child would be able to be involved and/or compete in wheelchair sports. For children who were currently participating in wheelchair sports, the parents mentioned that BoNT-A treatment was helpful in increasing range of motion in the muscle limbs, which enabled their child to engage in sports, such as sit on a horse. Although there were 35% of parents who identified that their child is currently participating in sports, such as horseback riding or sledge hockey, this was not the main outcome goal for treatment. Parents often identified improving range of motion and providing comfort to the child as the primary goal of BoNT-A treatment.

The initial inventory of goals in the context of botulinum toxin has been modified and updated based on these study findings. The goals identified and discussed by parents have been summarized and catalogued into the ICF domains of body structure and function, activity, and participation (see Additional file 2, PDF document: Inventory of Goals in the Context of Botulinum Toxin A Treatment). Parents identified sleep with few disturbances as a body structure / function goal and sit comfortably with good posture as an activity goal that were missing from the inventory. The therapists have also provided feedback, such as categorizing goals into certain ICF domains. The therapists provided three participation goals that were not listed in the initial inventory, in particular, recreational, school, and religious activities.

### Types of goals raised by parents

**Fig. 2:**
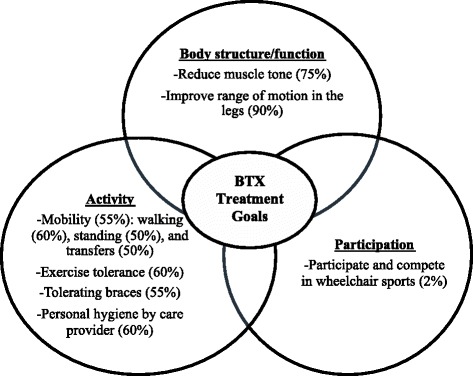
Main goals from BoNT-A treatment selected by parents

All treatment goals were identified and set by parents following a discussion with the healthcare providers (Table 2). The main goals identified were to reduce muscle tone (75%) and improve range of motion in the legs (90%). Some parents generally identified improving range of motion in the legs, but did not specify whether they wanted BoNT-A treatment to improve range of motion in the hips, knees or ankles. Among 14 children diagnosed with CP, classified as MACS Levels IV or V, there were only 3 parents who indicated improving range of motion in the arms as a goal for their child. Parents also identified engagement in functional activities, such as mobility (55%), specifically walking (60%), standing (50%), and transfers (50%). Some other functional activities that parents identified were exercise tolerance (60%), tolerating braces (55%), and use of equipment such as a walker (8%), wheelchair (5%), and stander (9%). There were 12 subjects whose parents identified hygiene to be a main goal, including changing diapers and toileting with the assistance of a care provider. There was only one child (9 years old, GMFCS level IV) who was able to conduct personal hygiene activities independently. There were some children whose parents identified participation goals including being able to participate and compete in activities (4%) and in wheelchair sports (2%). Figure 2 provides a summary of the main goals of BoNT-A treatment that were identified by parents.

**Table 2:** Goals for botulinum toxin A treatment according to International Classification of Functioning, Disability and Health component domain [6]

Specific goals identified according to ICF component domain	Identified goals	
	*N* = 20	%
Body structure and function		
Tone	15	75
To maintain and improve range of motion		
Legs	18	90
Hip	8	40
Knees	5	25
Ankles	5	25
Arms	6	30
Shoulder	3	15
Elbow	6	30
Wrist	3	15
Fingers	4	20
Drooling	3	15
Pain	6	30
Bone Health	3	15
Activity		
Mobility		
Walking	11	55
Standing	12	60
Transfers	10	50
Dressing	9	45
Shoes and socks	2	10
Time taken for dressing	8	40
Eating	3	15
Hygiene	11	55
By patient	1	5
By care provider	12	60
Exercise tolerance	12	60
Manipulating switches	4	20
Hand function	5	25
Tolerating braces	11	55
Use of equipment		
Walker	8	40
Wheelchair	5	25
Stander	9	45
Participation		
Being able to participate and compete	4	20
E.g. wheelchair sports	2	10

Abbreviation: ICF, International Classification of Functioning, Disability and Health

Most children received BoNT-A treatment after the interview. We reviewed the child’s medical charts to document the changes that were observed from BoNT-A injections, which were categorized as positive, negative, or neutral changes (Table 3). The changes that were observed by the healthcare professional team were similar to the goals raised by parents, such as reduced muscle tone, increased range of motion, and ease of caregiving such as dressing and personal hygiene.

**Table 3:** Description of BoNT-A injections in each child

Participant	GMFCS Level	Number of BoNT-A injections before interview	Number of BoNT-A injections after interview	Area of BoNT-A injections	Changes after BoNT-A injections	Outcome
1	4	6	5	Lower and upper extremities	Ease of dressing	Positive
					Able to sit more comfortably	
					Able to bear weight when transferring positions	
					Able to use assistive equipment	
2	4	1	0	Lower extremity	No change	Negative
3	4	1	1	Lower extremity	No change	Negative
4	4	5	7	Lower extremity	Able to stand straighter	Positive
					Able to sit in a wheelchair	
					Better sleep	
5	4	10	6	Lower extremity	Able to sit in a wheelchair	Positive
					Able to walk with a walker	
6	4	7	5	Lower and upper extremities	Ease of diapering	Positive
					Able to tolerate braces	
7	4	10	1	Lower extremity	Reduced muscle tone	Positive
					Decreased feeling of generalized pain	
					Better sleep	
8	4	8	4	Lower extremity	Reduced tone	Positive
					Able to use a stander	
9	4	11	1	Lower extremity	No change	Negative
10	4	< 3^a^	1	Lower extremity	Able to use assistive devices for walker	Positive
					Ease of transfers in position	
11	5	1	3	Lower and upper extremities	Ease of dressing	Positive
					Better sleep	
12	5	1	0	Lower extremity	No change	Negative
13	5	5	7	Lower extremity	Ease of standing with a stander	Positive
					Better sleep	
14	5	15	4	Lower extremity	Reduced tone	Positive
					Able to use a walker	
					Able to participate in horseback riding	
15	5	8	2	Lower extremity	Increased range of motion	Positive
					Ease of personal hygiene, including diapering and bathing	
					Ease of dressing	
16	5	8	6	Lower extremity	Personal hygiene	Positive
					Ease of dressing and transfers	
17	5	1	1	Lower extremity	Not documented	Neutral
18	5	7	4	Lower and upper extremities	Increased range of motion and stretching	Positive
					Ease of diapering	
19	5	13	7	Lower and upper extremities	Ease of dressing and bathing	Positive
					Able to participate in swimming	
20	5	< 3^a^	< 3^a^	Lower extremity	Not documented	Positive

^a^Exact frequency could not be retrieved at the time of the study

## Discussion

The objective of this study was to further refine an inventory list of goals based on the goals that parents have for their child with CP during BoNT-A treatment. This study also reports how an inventory of goals could be used as a tool to facilitate a discussion on setting goals between healthcare professionals and parents of children with CP assessed for BoNT-A treatment. Although all parents selected goals in the inventory, most parents found it challenging to identify goals that they thought would be appropriate in the context of BoNT-A treatment. Some parents were focused on “fixing” their child and wanted their child to achieve all of the goals listed in the inventory. This finding suggests that the process of setting goals could be framed using the ICF model in order to encourage parents to set goals that considers all aspects of the child’s life. After selecting the goals from the inventory list, parents were encouraged to provide additional goals that were not listed. Parents identified one body structure / function goal and one activity goal that were missing from the inventory list. The literature on goal setting also reflects this finding in which there is a wide variability in how parents perceive the goal setting process [22, 23]. Some parents wanted to be actively involved and prioritized their goals while other parents simply wanted the healthcare professionals to take responsibility and identify goals [22, 23]. It is important for healthcare professionals to explore and identify how parents perceive their role in the goal setting process.

There were more than 75% of parents who indicated that they wanted BoNT-A treatment to assist in the management of body structure and function, specifically reduction in muscle tone.

Similar parent-identified goals have been reported in other studies in which the parents focused on improving the functional limitations of the child [18, 19]. In a qualitative study, Schiariti et al. (2014) conducted 10 semi-structured interviews with parents and children (mean age 10 years 6 months, range 4-16 years) diagnosed with CP, with 7 children classified as GMFCS levels I-III and 3 children classified as GMFCS levels IV-V. All participants described impaired body structures, specifically the lower extremity, as a goal they would like to improve functioning on. The caregivers also reported goals regarding mobility and self-care, including assistance with completion of daily tasks such as dressing and personal hygiene [18]. Parents often experience difficulties in self-care tasks when caring for their child with CP, and would like BoNT-A treatment to facilitate ease of care.

Most parents did not mention goals related to participation until they were presented with the inventory list of goals. Parents would question whether their child could engage and attain participation goals due to their child’s motor abilities. Previous studies have shown that children and adolescents with CP are more likely to engage in informal activities, such as listening to music, watching TV, or visiting someone’s house compared to formal activities, such as recreational sports [20, 21]. In an informal discussion with the therapists, three new goals were identified in the participation domain, which included school activities, religious activities, and recreational activities, such as swimming, biking, and community groups. During the goal setting discussion, therapists should prompt families to consider different types of participation goals and share information to assist in reaching these goals. The inventory of goals can be used as a standardized tool for healthcare professionals to provide resources and suggestions for parents to engage their child in activities and participation. Some parents may require more information and choices in the types of goals that they could set with therapists, before they can become actively engaged in the goal setting process [22, 23]. The level of parental involvement in the goal setting process may depend on parents’ knowledge, needs, and expectations, which should be discussed with the therapists during treatment [23].

### Process of goal setting

Goal setting is an integral aspect of family-centred services in order to establish a partnership between parents and healthcare professionals [24–26]. When parents are involved in setting goals, they are informed about their child’s treatment plans and are motivated to encourage their child to achieve the identified goals [27, 28]. Parents also feel satisfied with the healthcare services that they receive because they had the opportunity to contribute to the treatments plans by sharing their needs, values, and beliefs. However, the implementation of goal setting in clinical settings is not standardized in rehabilitation services [29, 30]. Families of children with CP may have different needs and goals depending on the GMFCS level of the child. For example, parents of children and adolescents who use wheeled mobility often express needs in equipment and community activities [31].

The process of goal setting should be an ongoing collaborative discussion between the families and the therapists. There are several steps involved in the process setting goals, formulating action plans, and achieving health outcomes. Initially, a partnership is built where parents would share their story in order for the therapists to have a clear understanding of family needs and desires. The therapists would facilitate the discussion by asking questions that address the family priorities and intentions during treatment [30]. Parents would identify goals that are meaningful to their life, which should be clearly defined and documented [30]. The ICF-CY model may be appropriate to categorize and frame parent-identified goals [32]. The treatment process for a child with CP can be complex, and the domains of body structure/function, activity, and participation as well as the environment and personal factors are important components that may influence the family’s engagement and motivation during treatment.

An inventory of goals based on the ICF framework can be used as a tool to facilitate and document the goal setting process. A literature review conducted by Siebes et al. identified a need for a comprehensive family needs inventory for children with disabilities, families, and healthcare professionals in pediatric rehabilitation [19]. A family needs inventory was developed to assess family needs for children with physical disabilities [33]. There were more than 50% of parents who identified that they required information about development, treatment, and services for their child [33]. Parents often have difficulties identifying meaningful goals, and require the knowledge and skills on how they should set goals with healthcare professionals to determine an appropriate intervention [22, 34]. Specifically, parents of children with CP, classified as GMFCS level IV or V, require information on how to set meaningful goals and establish realistic expectations for the child’s ability to participate in activities. An inventory of goals may be beneficial as an informative tool for parents to better understand their child’s diagnosis and treatment in order to identify appropriate treatment goals with healthcare professionals.

### Study limitations

A limitation of this study is the lack of standardization in setting goals during the clinic appointment. During a busy clinic appointment, the goals were only identified by parents, and the goals raised by the therapists and clinicians were not documented. Additionally, all of the children and adolescents had cognitive impairments and did not participate in identifying goals in this study. Current evidence suggests that children with CP are able accomplish self-identified goals to the same extent as parent-identified goals [17]. For children with CP who are unable to verbally communicate, the use of alternative communication methods, such as eye-gaze speech devices, may be helpful to incorporate the child’s perspective during the goal setting process [36, 37]. The inventory of goals in the context of BoNT-A treatment should be modified to clearly document whether the parents, healthcare professionals, or child are selecting the goals. All participants were recruited from the Spasticity Management Clinic at McMaster Children’s Hospital and the inventory of goals may not be a comprehensive list of all goals that parents have for their child during BoNT-A treatment. Furthermore, the age of the participants ranged from 5 to 18 years, and the inventory of goals may not include the goals from parents of children with CP and younger than 5 years old. However, this study provides us with a better understanding of the most common goals elicited by parents when their child receives BoNT-A treatment. The use of this inventory of goals in other clinical settings may be a beneficial tool to facilitate the goal setting discussion as well as encourage sharing of information and resources by therapists. Parents can also be provided with the option to identify goals that are missing from the inventory during the goal-setting discussion.

### Implications for clinical practice

This cross-sectional observational study allowed us to review our clinical practice in family-centred care and goal setting with children with CP, GMFCS levels IV and V, and families. In the Spasticity Clinic at McMaster Children’s Hospital, setting goals is already integrated into the clinic appointment. The development and use of an inventory of goals may be helpful in clearly documenting the goals identified by parents. Currently, the inventory of goals has been structured for parents to complete at the beginning of a clinic appointment. The goals have been categorized into the ICF domains of body structure/function, activity, and participation. Therapists can clearly outline the parent-identified goals in an initial appointment, monitor the child’s progress on those goals, and re-evaluate the appropriateness of those goals based on family needs at follow-up appointments.

### Implications for future research

We modified and updated an inventory of goals for children with CP, classified as GMFCS levels IV or V. Since the inventory of goals is a work in progress, future research should focus on better understanding parents’ perspectives and how they perceive their role in the goal setting process. A future study is needed to conduct qualitative interviews in order to better understand parents’ past experiences of BoNT-A treatment, the information parents may require about setting relevant and meaningful goals during treatment, and parents’ perceived usefulness of the inventory of goals. Healthcare professionals and families can also provide feedback on the current inventory of goals as it continues to be further developed and refined.

## Conclusion

The results of this study suggests that an inventory of goals in the context of botulinum toxin treatment for children and adolescents with CP, GMFCS levels IV and V, may be beneficial as a tool to facilitate the goal setting process. An inventory of goals in the context of BoNT-A treatment could help create a common language for families and healthcare professionals to identify goals that are relevant and meaningful to the family based on their values, needs, and beliefs. The goal setting discussion could be used as an opportunity for healthcare professionals to provide information and resources to encourage families to engage their child in activities and participation.

## Additional files

Additional file 1: *Inventory of Goals in the Context of Botulinum Toxin A Treatment.* An initial inventory of goals was developed by a special interest group at the national botulinum toxin conference held in January 2014 (Montreal, Canada). There were 16 items categorized into three domains based on the ICF framework: body structure/function, activity, and participation. In this study, parents were asked to select items from this initial inventory that they would like their child to accomplish as goals after botulinum toxin treatment. Parents were also asked to provide additional goals that were not listed in the inventory. (PDF 165 kb)

Additional file 2: *Inventory of Goals in the Context of Botulinum Toxin A Treatment (modified).* A revised version of the inventory has been created based on study findings. Parents identified one body structure/function goal and one activity goal, which were not listed in the draft inventory list of goals. The therapists identified three participation goals. Feedback from the research team and therapists led to the re-categorization and rephrasing of goals. The revised ad updated inventory list of goals is comprised of 20 goals categorized into the ICF domains of body structure and function, activity, and participation. (PDF 207 kb)

## Abbreviations

BoNT-A: Botulinum toxin treatment
CFCS: Communication Function Classification System
CP: Cerebral palsy
GMFCS: Gross Motor Function Classification System
ICF: International Classification of Functioning, Disability and Health
MACS: Manual Ability Classification System
