# Supplementary material for: Development of an inventory of goals using the International Classification of Functioning, Disability and Health in a population of non-ambulatory children and adolescents with cerebral palsy treated with botulinum toxin A
Source: BMC Pediatr. 2018 Jan 4;18:1. doi: 10.1186/s12887-017-0974-x (PMC5755464; doi:10.1186/s12887-017-0974-x)
Supplement: Supplementary file 1 — Inventory of Goals in the Context of Botulinum Toxin A Treatment. An initial inventory of goals was developed by a special interest group at the national botulinum toxin conference held in January 2014 (Montreal, Canada). There were 16 items categorized into three domains based on the ICF framework: body structure/function, activity, and participation. In this study, parents were asked to select items from this initial inventory that they would like their child to accomplish as goals after botulinum toxin treatment. Parents were also asked to provide additional goals that were not listed in the inventory. (PDF 165 kb) [file 12887_2017_974_MOESM1_ESM.pdf]

McMaster Children's Hospital
HAMILTON HEALTH SCIENCES

McMaster University
Department of Pediatrics

Participant's GMFCS Level: ________

Inventory List of Goals

Body Structure / Function	Activity	Participation
☐ Tone<br><br>**Range of Motion**<br>☐ Legs<br>☐ Hip<br>☐ Knees<br>☐ Ankles<br><br>☐ Arms<br>☐ Shoulder<br>☐ Elbow<br>☐ Wrist<br>☐ Fingers<br><br>☐ Drooling<br><br>☐ Pain<br><br>☐ Bone Health	☐ Mobility<br>☐ Walking<br>☐ Standing<br>☐ Transfers<br><br>☐ Dressing<br>☐ Shoes and socks<br>☐ Time taken for dressing<br><br>☐ Eating<br><br>☐ Hygiene<br>☐ By patient<br>☐ By care provider<br><br>☐ Exercise tolerance<br><br>☐ Manipulating switches<br><br>☐ Hand function<br><br>☐ Tolerating Braces	☐ Use of equipment<br>☐ Walker<br>☐ Wheelchair<br><br>☐ Being able to participate and compete<br>☐ e.g. wheelchair sports
