# Supplementary material for: Development of an inventory of goals using the International Classification of Functioning, Disability and Health in a population of non-ambulatory children and adolescents with cerebral palsy treated with botulinum toxin A
Source: BMC Pediatr. 2018 Jan 4;18:1. doi: 10.1186/s12887-017-0974-x (PMC5755464; doi:10.1186/s12887-017-0974-x)
Supplement: Supplementary file 2 — Inventory of Goals in the Context of Botulinum Toxin A Treatment (modified). A revised version of the inventory has been created based on study findings. Parents identified one body structure/function goal and one activity goal, which were not listed in the draft inventory list of goals. The therapists identified three participation goals. Feedback from the research team and therapists led to the re-categorization and rephrasing of goals. The revised ad updated inventory list of goals is comprised of 20 goals categorized into the ICF domains of body structure and function, activity, and participation. (PDF 207 kb) [file 12887_2017_974_MOESM2_ESM.pdf]

Inventory List of Goals
in the Context of Botulinum Toxin A Treatment

Participant's GMFCS Level: ________

Please read the following and mark the boxes beside the description that best represents the body structure and function, activity, and participation that you would you like your child to achieve after botulinum toxin treatment.

I would like the botulinum toxin treatment to help my child to…

Body Structure / Function	Activity	Participation
**<u>Body Structure</u>**<br><br>**Improve Range of Motion**<br>□ In the legs<br>□ and straighten the hips<br>□ and straighten the knees<br>□ and straighten the ankle<br><br>□ In the arms<br>□ and raise the shoulders<br>□ and straighten the elbows<br>□ and straighten the wrists<br>□ and bend and flex the fingers<br><br>**<u>Function</u>**<br>□ Reduce overall muscle tone<br><br>□ Reduce the amount of drooling<br><br>□ Reduce the feeling of generalized pain<br><br>□ Increase bone health and strengthen bones<br><br>□ Sleep with few disturbances	□ Increase mobility<br>□ and walk for longer distances<br>□ and stand for longer periods of time<br>□ Facilitate ease of transfers in position (ex. from bed to chair) by care provider<br><br>□ Sit comfortably and with good posture<br><br>□ Be able to use assistive equipment<br>□ and use a walker<br>□ and use a wheelchair<br>□ and use a stander<br><br>□ Tolerate braces<br>□ and wear braces for a longer period of time<br><br>□ Tolerate exercise<br>□ and be able to do stretching exercises<br><br>□ Manipulate and use small objects with hands (e.g. writing supplies such as a pencil, light switches)<br><br>□ Manage personal hygiene (e.g. diapering, toileting)<br>□ By patient<br>□ By care provider<br><br>□ Change clothes<br>□ and facilitate ease of dressing (e.g. socks, pants)<br>□ By patient<br>□ By care provider<br>□ and reduce the time taken to put on clothes<br>□ By patient<br>□ By care provider<br><br>□ Eat meals<br>□ and provide easier use of utensils<br>□ and reduce the time taken to eat meals	□ Be able to participate and compete<br>□ In wheelchair sports<br><br>□ Recreational activities<br>□ Swimming<br>□ Biking<br>□ Community groups<br><br>□ School activities<br><br>□ Religious activities
